# Temporal and spatial distribution of human cryptosporidiosis in the west of Ireland 2004-2007

**DOI:** 10.1186/1476-072X-8-64

**Published:** 2009-11-24

**Authors:** Mary Callaghan, Martin Cormican, Martina Prendergast, Heidi Pelly, Richard Cloughley, Belinda Hanahoe, Diarmuid O'Donovan

**Affiliations:** 1 Environmental Change Institute, National University of Ireland, Galway, Ireland; 2 Department of Medical Microbiology, Galway University Hospital, Newcastle Road, Galway, Ireland; 3 Department of Public Health, Health Service Executive West, Merlin Park, Galway, Ireland

## Abstract

**Background:**

Cryptosporidiosis is increasingly recognised as a cause of gastrointestinal infection in Ireland and has been implicated in several outbreaks. This study aimed to investigate the spatial and temporal distribution of human cryptosporidiosis in the west of Ireland in order to identify high risk seasons and areas and to compare Classically Calculated (CC) and Empirical Bayesian (EB) incidence rates. Two spatial scales of analysis were used with a view to identifying the best one in assessing geographical patterns of infection. Global Moran's I and Local Moran's I tests of autocorrelation were used to test for evidence of global and local spatial clustering.

**Results:**

There were statistically significant seasonal patterns of cryptosporidiosis with peaks in spring and an increasing temporal trend. Significant (p < 0.05) global spatial clustering was observed in CC rates at the Electoral Division (ED) level but not in EB rates at the same level. Despite variations in disease, ED level was found to provide the most accurate account of distribution of cryptosporidiosis in the West of Ireland but required spatial EB smoothing of cases. There were a number of areas identified with significant local clustering of cryptosporidiosis rates.

**Conclusion:**

This study identified spatial and temporal patterns in cryptosporidiosis distribution. The study also showed benefit in performing spatial analyses at more than one spatial scale to assess geographical patterns in disease distribution and that smoothing of disease rates for mapping in small areas enhances visualisation of spatial patterns. These findings are relevant in guiding policy decisions on disease control strategies.

## Background

*Cryptosporidium *species cause gastrointestinal infection in humans and animals and are now the commonest protozoan parasites associated with gastroenteritis in Ireland [1]. The genus comprises many morphologically similar species that are distinguished by genotype and by host range [2]. The two species most relevant to human health are *C. hominis*, which is a pathogen of humans and is not associated with animal infection, and *C. parvum *which has a broad mammalian host range including humans [2,3].

The epidemiology of sporadic (non-outbreak associated) cases is poorly understood. Infection varies spatially, with *C. parvum *more prevalent in rural areas, reflecting contact with farm animals. In the United Kingdom, socioeconomic status was found to be associated with risk factors for infection such as foreign travel and swimming pool use [4]. Type of water supply [5] and water treatment methods are important risk factors. In humans both species are associated with acute diarrhoea with or without additional gastrointestinal symptoms following an incubation period of 7 to 14 days [6,7]. The condition is self-limiting in otherwise healthy people, but can cause intractable diarrhoea in patients with impaired immune function. The infection is transmitted by the faecal-oral route, either by direct person-to-person contact or indirectly through contamination of food or water. Several large outbreaks of human cryptosporidiosis have been reported, often associated with contaminated water supplies. The prevention and control of waterborne outbreaks of cryptosporidiosis is difficult because the oocysts of the protozoan cryptosporidium are not inactivated by chlorination at levels used in drinking water. As chlorination is not effective in destroying the oocysts, the protection of drinking water from contamination is dependent on source protection (limiting animal and human faecal contamination of water sources) and on removal or inactivation of cryptosporidium oocysts in the water treatment process. Water that is fully compliant with accepted bacteriological standards (absence of *E. coli*, general coliform bacteria, enterococci and *C. perfringens*) may contain viable cryptosporidia. Conventional water treatment processes including chemical coagulation, flocculation, sedimentation, filtration and disinfection successfully remove the majority of microorganisms in raw water which are a concern to public health [8], but these methods do not always remove cryptosporidium. Advances in water treatment that successfully remove cryptosporidium when the systems components are intact and operating correctly include microfiltration and ultrafiltration, and the most effective method to inactivate any cryptosporidium remaining after filtration is ultraviolet treatment [8], which requires that the treated water is of low turbidity. However, treatment methods alone cannot solve the problem; protecting water supplies and monitoring water quality are crucial.

When investigating spatial disease patterns it is not clear what the most appropriate spatial scale (areal unit) is [9]: this is the phenomenon of the Modifiable Areal Unit Problem (MAUP) where spatial patterns in disease distribution may change with a change of the spatial scale of analysis. There are two main problems associated with MAUP: the zone effect and the scale effect. It is important to acknowledge the role that the zone of analysis has on health data when constructing and producing maps [10]. The zones are the basic building blocks of maps, which are usually countries or states, with boundaries which can be epidemiologically arbitrary and can be changed. Consequently, any patterns observed across zones may be more a function of the zone boundaries, rather than of the spatial distribution of the values themselves [11]. Scale is also not readily understood in relation to disease mapping. At different scales, the same data can produce completely different results [10,12]. Patterns at one scale may be highly evident, but at another may not even exist [13,14]. As a result correlations between variables and outcomes can seem to be reversed at different scales. This can lead to considerable confusion when hypotheses rejected at one level are accepted at another. Because of such confusion, it is good practice if disease and health care investigations are carried out at several different geographic levels [15]. With this in mind it is essential that unbiased maps of disease occurrence give a realistic portrayal of the situation under investigation with minimized error. A number of solutions to MAUP have been suggested. One such solution could be to re-aggregate the data to another set of zones [14]. However this is almost always impossible due to the limited availability of higher resolution data and difficulties in assessing the ecological fallacy associated. Selecting appropriate class intervals and display colours is another possible solution [11]. The problem could be avoided by using continuous shading or gradation of colour. However, it is widely recognised that the effects of MAUP are difficult to control and that there is no definitive solution [11], therefore it is necessary to understand its effects, and become aware of its existence and impact [16].

Measures of incidence rates of disease patterns in small areas have limitations: as populations are dynamic, mapping and statistical comparisons with different variances need to be conducted; and rates in areas of low population usually have high variances but are more unstable than those from areas of high population [17,18]. Low population areas are often rural, and cover large areas, giving undue visual impressions that may be unreliable due to higher variances.

Traditionally, disease mapping has been dominated by the use of the Standardized or Classically Calculated (CC) Incidence/Mortality rates or measured relative risk to display geographical variability. Although mapping standardized rates is a useful exploratory device, which has been put to considerable use in medical geography and epidemiology, this method is usually unable to deal effectively with diseases affecting small numbers of people. In small number diseases, disease rates tend to be extremely unreliable because of the small numbers upon which it is based. One method of getting around this problem is to use "smoothed" estimates of disease [19] such as the Empirical Bayesian (EB) method. The Bayesian approach is a form of statistical estimation, where observed data and prior knowledge on parameters of interest are considered when estimating their values.

The objectives of this study were to describe the spatial and temporal distribution of cryptosporidiosis cases reported to the surveillance system in the Irish Health Service Executive (HSE) Western Area in order to identify areas with high rates, to identify the appropriate spatial scale for mapping of the infection in this area, and to compare Classically Calculated (CC) and Empirical Bayesian (EB) incidence rates.

**Figure 1:**
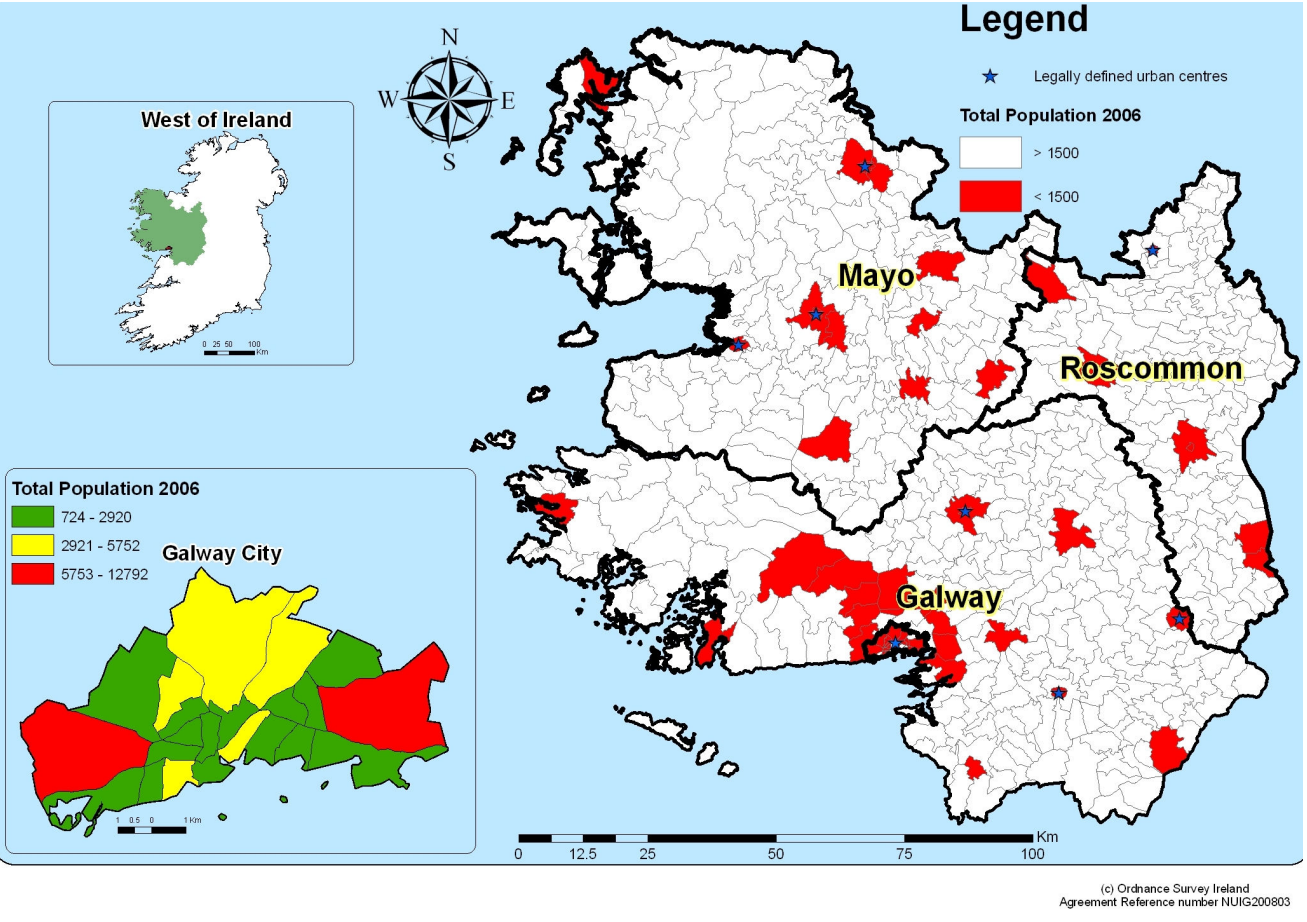
**Urban Centres in the West of Ireland 2006**.

Figure 1 shows the local authority boundaries which are nearly all co-terminous with most Local Health Office boundaries, and urban and rural EDs as defined by the Central Statistics Office [20]. Local Health Offices are the administrative units of the Irish health service for Primary Community and Continuing Care, which currently include Environmental Health services [21].

## Methods

### Data collection and manipulation

Cryptosporidiosis is a notifiable disease in Ireland in all age groups since 1^st ^January 2004 [1]. Before then, a case was only notifiable if it was cited as a cause of gastroenteritis in a child younger than two years.

The study area is the Health Services Executive (HSE) Western Area located on the west coast of Ireland encompassing all of County Galway and Galway City, County Mayo and County Roscommon. The total population for this area is approximately 414, 277 [22] spread across 14, 280 km^2^. Spatial analysis was carried out at the district (n = 30) and electoral division (ED) (n = 498) levels. Surveillance data on cryptosporidiosis collated by the Public Health Department at HSE West and the microbiology laboratory at Galway University Hospital for the years 2004-2007 form the basis of this study. The study population included all laboratory confirmed cases of cryptosporidiosis with addresses in the area.

Personal identifiers of the patients were deleted before the database was released to the investigators. The variables available for analysis included age, sex and address at diagnosis. Address at diagnosis was used as the spatial locator for all cryptosporidiosis cases. From the original dataset of 593 confirmed cases, 569 (95%) cases were used in the analysis. It was possible to match 365 (64%) addresses to ED level without revisions: after spelling revisions this was increased to 569 addresses. It was not possible to match 24 (4%) of the original addresses due to missing information, invalid addresses or addresses located outside the study area: these were excluded from the study.

The GeoDirectory was obtained from An Post GeoDirectory Limited [23]. GeoDirectory is a complete database of unique, verified addresses of all buildings in the Republic of Ireland together with a precise Geocode. The GeoDirectory was used to assign each disease case to its implicit location to display cases on a map. The total observed number of cases of cryptosporidiosis was taken from the annual publications of the HSE and Health Protection Surveillance Centre (HPSC) on the epidemiology of cryptosporidiosis in Ireland, and the total population counts were sourced from the 2006 Irish census data [22].

### Statistical Analyses

The disease standardized incidence was calculated by dividing the observed cases by the expected cases for each spatial unit. These rates were expressed as percentages with those higher than 100% having above average national rates and those below 100% having below average national rates. Cryptosporidiosis in Ireland is essentially a small numbers disease with many EDs having fewer than 2 cryptosporidiosis cases. Global and local Empirical Bayesian smoothing was performed at both spatial levels.

Temporal patterns were displayed by plotting the monthly number of notified cryptosporidiosis cases. Months were grouped into their respective seasons with February, March, April as spring, May, June and July as summer, August, September and October as autumn and November, December and January as winter. To test whether there was heterogeneity between the different months, we used a χ^2 ^test for trend taking into account the differences in the number of days in the months. In a second analysis testing for the presence of seasonality, we used Edward's test for a twelve month period.

### Computation of measures of spatial clustering

Global Moran's I and Local Moran's I were computed for cryptosporidiosis rates using tools within Arc toolbox. Z scores were used to indicate statistical significance and maps were produced.

### Cartographic Displays

Choropleth maps were produced to examine and explore the differences in spatial distribution between the different cryptosporidiosis incidence rates at the different geographical scales. ESRI's ArcView 9.2 was used to manipulate data and produce the maps. Mapping intervals were assigned using Manual classifications. The groupings assigned to EB rates matched those assigned to CC rates. Clustering of rates were measured using Local and Global Moran's I.

## Results

### Temporal distribution

**Figure 2:**
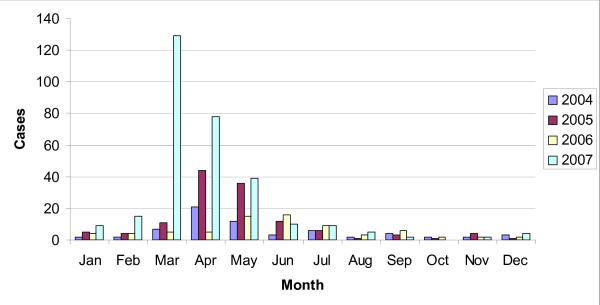
**Distribution of cryptosporidiosis each month over four years**.

There were seasonal patterns in the distribution of cryptosporidiosis with the highest number of cases occurring during the late spring months of March and April. Figure 2 shows the distribution of cryptosporidiosis each month over four years. Table 1 shows the observed frequency and expected frequency of disease cases per month if there were no seasonality, taking into account the difference in number of days in each month. A trend of seasonality was observed with cryptosporidiosis cases occurring more frequently in spring and summer months compared to autumn and winter (χ^2 ^for trend 560.133 df 3; p = 0.000). Edward's test showed a seasonal pattern, which peaked in March (p = 0.000). The data includes a major outbreak in early 2007 due to contamination of the water supply in Galway City. Typing of isolates was only possible on a small proportion of the cases, as there is no cryptosporidium reference laboratory in Ireland: the data refer to both *C. hominis *and *C. parvum*

**Table 1:** Monthly distribution of cryptosporidiosis

	n = 569	
	
	Observed	Expected
Jan	20	48.3
Feb	25	43.6
Marc	152	48.3
Apr	148	46.8
May	102	48.3
Jun	41	46.8
Jul	30	48.3
Aug	11	48.3
Sept	15	46.8
Oct	5	48.3
Nov	10	46.8
Dec	10	48.3
		
χ^2 ^11 df	681.94	
*p*-value	0.000	

### Spatial distribution

**Figure 3:**
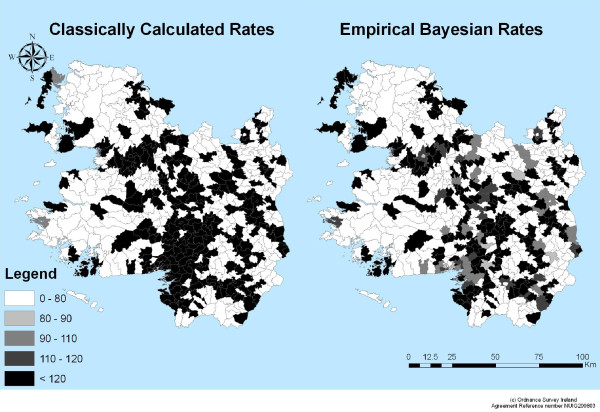
**Distribution of Classically Calculated cryptosporidiosis Incidence Rates at the ED and District level**.

**Figure 4:**
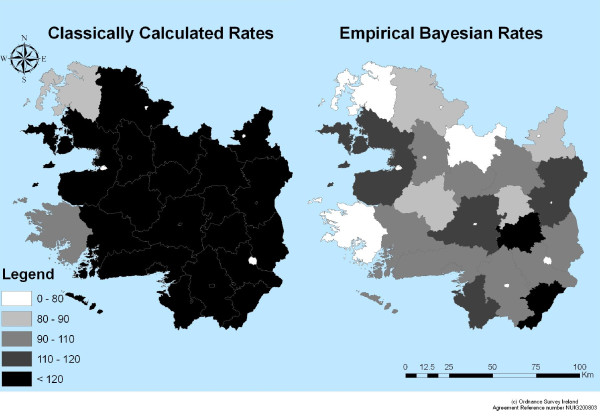
**Distribution of Empirical Bayesian cryptosporidiosis Incidence Rates at the ED and District level**.

Maps showing the geographical distribution of CC and EB incidence rates are shown in Figures 3 and 4. Visually, at the ED level, high CC rates appear to be concentrated around the main urban centres in the West of Ireland including Galway City, Castlebar, Tuam, Loughrea and Roscommon town (CC>120). At the district level, the majority of districts are seen to have CC incidence rates above the national average. There are also a large number of EDs from which no cases of cryptosporidiosis were reported (Figure 3). In comparison, the EB rates produced more distinct patterns of disease distribution highlighting potential clusters around the West of Ireland. High EB rates appeared to be concentrated in rural areas. There are also only two districts which have an EB rates above the national average (Figure 4).

### Measures of spatial clustering of cryptosporidiosis rates

Using inverse distance weights, there was a significant global autocorrelation of CC cryptosporidiosis rates at the ED level (p < 0.01). EB rates at the ED level were found to be random and not significant. At the district level, both CC and EB incidence rates produced a weak negative result which indicates a random pattern with no global autocorrelation (p < 0.05). Local Moran's I statistic was also used to examine small scale or local auto correlation within the datasets at the different geographic levels. CC cryptosporidiosis rates are mostly dispersed throughout counties Galway, Mayo and Roscommon, though there is some clustering of higher CC cryptosporidiosis rates in the east and south east of Galway City and in the south of County Roscommon. Similar to CC calculated rates of cryptosporidiosis, EB cryptosporidiosis rates were mainly dispersed throughout the study area. Clustering of EB cryptosporidiosis rates is weaker (values of 1-6) than that of CC calculated rates. The strength of the local Moran's I values for CC cryptosporidiosis rates are notably weaker at district level to that of the values at ED level indicating that CC cryptosporidiosis rates are generally dispersed throughout the West of Ireland with little evidence of clustering. Stronger positive values of Moran's I in districts with higher than average cryptosporidiosis rates are located to the east and south east of Galway City and also in the north and North West of County Mayo. However, the Moran's I values are quite low, indicating that there may only be a slight clustering in these areas. The Local Moran's I statistics for EB cryptosporidiosis rates in the West of Ireland, at district level also indicate very little spatial autocorrelation exists in EB incidence rates.

## Discussion

### Geographical and temporal patterns of cryptosporidiosis distribution

The increasing temporal trend in observed cryptosporidiosis cases could be a result of improved surveillance in Ireland. Before January 2004, routine testing and notification of cryptosporidiosis was not carried out in all age groups. On the 1^st ^January 2004, a new list of notifiable infectious diseases came into place [24] when cryptosporidiosis became a notifiable disease in all age groups and a requirement was introduced for confirmed cases to be reported. There was also an outbreak of cryptosporidiosis in Galway city in March 2007 which accounts for the notable increase in cases in that year [25].

A seasonal peak is observed in cryptosporidiosis in the late spring and early summer months. These peaks coincide with the lambing and calving season, and with the spreading of agricultural waste (slurry) as fertiliser. The Edwards test shows a monthly peak in cryptosporidiosis in March. This peak may be emphasized by the confirmed outbreak in March 2007. It is possible that heavy rainfall in the preceding winter may have increased flooding and subsequent water contamination as the lake that provides the water supply to the outbreak affected area was at its second highest level ever recorded just before the outbreak.

Although there was evidence of clustering of cryptosporidiosis at the ED level, there was no evidence of this at the district level possibly due to the scale effects of the MAUP. In relation to the CC incidence rates at the ED level, clustering of these cases tended to occur in areas with dense populations reinforcing the need for the EB incidence rates to smooth out disease incidence and reduce the variability in disease rates. Scale effects of MAUP and EB methods were found to have the greatest effect on the geographical distribution of the cryptosporidiosis rates.

### Smoothing of rates for mapping

EDs are the smallest spatial unit for which small area population statistics are currently available in Ireland and are therefore the smallest unit examined in this study. They have the smallest number of cases and the smallest populations, making rate calculations at this level more unstable and resulting in extreme values. In the West of Ireland, the average population of an ED is 832 (range 86-12,792) [22].

The EB approach to rate calculation differs from that of the CC method in that it has used prior knowledge or beliefs about parameters of interest to increase the stability of the most unstable results and regulate extreme values [11,26]. In this study, the majority of EDs have no observed cryptosporidiosis incidence, resulting in rates for these EDs below the national standardized rate. Addition or deletion of even just one or two events can result in drastic changes in the observed value [27]. The calculation of EB estimates for disease rates has been suggested as a means to stabilize extreme values [27]. When the observed count increases from zero, the effects of the EB smoothing can be seen. The rates calculated by the CC method produce more extreme and variable results than those calculated by EB methods (Figures 3 and 4). Overall, it can be seen that EB calculated rates exhibit lower values which are closer to the national average than those calculated by the CC method. It is apparent that CC rates are more variable and therefore more unstable, with the maximum CC rate recorded at over 30 times greater than the national average. This is a typical result, in that the population at risk and the number of observed cases are too small resulting in unreliable and highly variable rates [11]. This is not the case with the EB calculated rates: using this method has moderately lessened the small numbers problem resulting in more stable rates, which fluctuate around the national average.

Districts are the next spatial unit at which the data is examined in this study. Spatially, districts are larger than EDs which nestle into districts. There are 30 districts in the West of Ireland included in this study, of which 9 are classified as urban and 21 as rural. The average population of a district in the West of Ireland is 13,809 (range 1,600-72,414) [22].

At district level, calculating EB incidence rates produced more stable results as there was less variation in the data. At this level, only two districts had zero cases of cryptosporidiosis in comparison to a large number of EDs having zero cases of cryptosporidiosis. However due to the aggregation of the data to this level, there was a loss in spatial information. There was an average of 19 cases of cryptosporidiosis per district (range 0-108) [22]. There is clearly a large amount of hidden variation in the data at district level. The small numbers problem can be seen at district level for the CC incidence rates but not for the EB incidence rates, indicating that at this level EB incidence rates are better able to deal with the small numbers issue.

### Choice of spatial scale of analysis

Through changing the level at which the data is examined from ED to District level, the observed changes in both sets of disease incidence rates can be seen as the scale effect of the MAUP. At the ED level the non-spatial distribution of disease rates is quite different to that of the district level. The different distributions of diseases at the different levels are only a product of the aggregated zones from which they are created and therefore any results obtained are conditional upon the zones on which they are set [11,28]. Consequently any patterns observed at each of the different levels may be as much a product of the zone boundaries as it is of the underlying distribution of rates.

The scale effects of MAUP can be notably seen at all levels in this study. Using the EB incidence rates as an example, at district level, 10 districts (33%) were found to have EB rates higher than that of the national average while at ED level, 195 EDs (39%) were found to have rates higher than that of the national average. The aggregation problem of MAUP is illustrated clearly here in that the variation in EDs changes or is lost when the data is aggregated to district or county level [10]. This example also highlights the problem of the ecological fallacy, in that it is implied that all individuals living in an area share the characteristics of that area [16,18] which is obviously not true. In this study both the EB and CC rates have been shown to illustrate very different distributions at ED and District level, but this study does not provide sufficient evidence for one approach over the other due to small numbers of the disease.

### Limitations

One limitation to this study was the relatively low number of cases of cryptosporidiosis. Low numbers causing high variability in rates may have introduced errors into the calculations and therefore have had an effect on statistical results of this study. A further limitation in the data was that there was a confirmed outbreak of cryptosporidiosis in County Galway in 2007. This data may have skewed the dataset as there were over twice as many cases recorded for this year than any of the other years under investigation. Exclusion of this data from the whole dataset was considered but it was felt that the effects of the small numbers problem would have been amplified further in doing so.

The extent to which the actual incidence and distribution of cryptosporidiosis is reflected in the data is also limited by complex potential disease ascertainment bias. Only laboratory confirmed cases are available for mapping. This represents those individuals who developed diarrhoea, who sought medical advice, from whom a specimen of faeces was submitted to the laboratory, and from whom a specimen was tested based on the practice of the receiving laboratory. The extent to which the number of laboratory confirmed cases under-represents actual cases and the potential for distortion of geographical distribution arising from ascertainment bias is poorly understood as we do not know how many people who were unwell did not attend their family doctor, and we do not know how many of those who attended did not have laboratory specimens sent.

## Conclusion

This study shows visual and statistical evidence of spatial clustering as well as significant seasonal patterns and increasing temporal trend in the distribution of cryptosporidiosis cases in the West of Ireland. This study showed that the ED level, although not ideal, is more appropriate than district level for mapping cryptosporidiosis rates in Ireland. However mapping at this level requires smoothing to reduce variability in disease rates. This study has clearly demonstrated the usefulness of Geographic Information Systems (GIS) in analyzing and exploring disease incidence. Its unique ability to integrate a large range of datasets in a common framework facilitating in the spatial and non spatial analysis of disease events adds a different dimension to disease analysis and surveillance. Through analyzing disease in a spatial format, trends and interrelationships may be revealed more easily than would be in tabular format [29]. Its efficiency in visualizing problems also allows policy makers to target resources more efficiently. GIS software is now more user friendly allowing a wider audience to make use of this powerful tool. Further studies to investigate risk factors in more detail could provide more information to inform risk assessments and control strategies.

## Competing interests

The authors declare that they have no competing interests.

## Authors' contributions

MCA was involved in research design, data collection, analysis and preparation of the manuscript. DOD and MCO were involved in conceptualization, design, execution and manuscript preparation. HP was involved in research design and manuscript preparation. RC and BH were involved in data acquisition and manuscript preparation. MP was involved in the conception and design of the project and in manuscript revision.
